# The Relevance of Metaphysics to Behavior Analysis

**DOI:** 10.1007/s40614-020-00277-5

**Published:** 2021-01-20

**Authors:** Julian C. Leslie

**Affiliations:** School of Psychology, Ulster University, Coleraine, Northern Ireland UK

**Keywords:** Metaphysics, Naturalism, Dualism, Monism, Private events

## Abstract

Behavior analysis takes a natural science approach to human and animal behavior. Some basic tenets are widely agreed in the field but it can be argued that some other assumptions are implicit in our approach and, if unexamined, may impair progress. Since the time of David Hume, there has been a strong Western philosophical tradition of naturalism and realism. Although behavior analysis has from the outset embraced pragmatism, features of naturalism are embedded in the metaphysics of science and thus have been imported into behavior analysis. Many versions of naturalism imply dualism, but this can be avoided without abandoning a naturalist–realist position either by adopting the historicist approach of Rorty, which suggests that apparently a priori truths are often merely conventions of a philosophical tradition, or by accepting Wittgenstein’s view that there are hinge statements that are fundamental to our thinking but are not propositional beliefs and do not entail dualism. As an alternative, we can adopt the metaphysical assumptions of monism, possibly starting from William James’s approach of neutral monism. Revising our metaphysical assumptions while retaining the pragmatism that is central to behavior analysis may enable us to engage more effectively with cognitive psychology, to develop stronger links with ecological psychology and other approaches that reject representationalism, and to move beyond the debate about the status of private events.

Metaphysics is an area of philosophy, not science. The term originated with Aristotle, although at that time applied more to what we now call physics (Hamlyn, 2005). In a broad sense, it concerns the nature of ultimate reality. Within contemporary metaphysics, one major concern is the study of that which is known “a priori,” and what can be deduced from what is so known. In Western philosophy since the time of Immanuel Kant, a-priori knowledge has been defined as knowledge independent of all particular experiences. As well as mathematical principles and tautological statements, this may include unstated presuppositions, perhaps inherent in the language we use. Although texts may briefly review defining features of science as well as providing some history of the discipline (e.g., Cooper, Heron, & Heward, 2020; Pierce & Cheney, 2017), an introductory account of behavior analysis typically begins without any review of metaphysical assumptions (cf. Catania, 2017), and that is typical of introductions to other sciences, such as branches of physics, chemistry, or biology. Instead, introductions to science are typically of the form: we study this domain of interest using these methods. . . . Thus, we might start a behavior analysis course by saying, “We study the behavior of humans and other animals using these methods. . . .”

The conventional—and usually unspoken—view of the scientist is that if the putative science produces a set of coherent findings with whatever methods it is using, then it is on its way, but Ghiselin (1997), an evolutionary biologist, argued forcefully that we cannot have science without metaphysics, and that without appropriate metaphysics we may make categorical errors and produce meaningless statements (Dennett [2013] also gave various examples of where scientists pursue pointless questions because they have failed to first examine the philosophical underpinnings). Ghiselin is best known for arguing that species are logical individuals rather than classes*,* and in his view the ontological or metaphysical error of treating them as classes leads to other serious errors*.* Baum (2017) extended Ghiselin’s approach to the science of behavior, and stated, inter alia, that behaviors should be conceived of as activities that are ontological individuals, that is, entities that have a beginning and an end and occupy a certain spatial location. Here I consider the possibility that dealing with some other metaphysical issues may help resolve issues in behavior analysis and its relationship with psychology that otherwise seem intractable.

It could be argued that Skinner (e.g., 1938, 1945, 1957, 1974; see Delprato & Midgley, 1992) dealt with metaphysical issues in general by developing a form of pragmatism that underpins the experimental analysis of behavior. Pragmatism is a philosophical stance wherein a proposition is true if it works satisfactorily, and statements or ideas that do not meet this criterion are rejected. Following Watson (see, e.g., Watson, 1930), Skinner maintained that the primary objective of a science of behavior is prediction and control of its subject matter. Zuriff (1980) extensively reviewed the form or forms of pragmatism presented in Skinner’s work, and found many examples where Skinner identified verbal behavior as true, and thus contributing to knowledge, where it leads to other behavior that is “effective,” “successful,” “useful,” etc., and noted that this pragmatic approach to knowledge is strongly related to the positivist philosophy of science Skinner took from Mach (1893). The philosophical principles of behavioral pragmatism were more explicitly stated by Barnes-Holmes (2000), building on the work of Barnes and Roche (1994). According to Barnes-Holmes (2000),

For the behavioral pragmatist all scientific talk participates in a single behavioral stream containing (a) the pragmatist's verbally stated goals, (b) the pragmatist's analytic talk about how to achieve them, and (c) the pragmatist's statement as to whether or not they have been achieved. *Such talk is a-ontological* in the sense that it is verbal behavior (i.e., it involves the dynamic and codefining interaction among stimulus and response functions). Verbal behavior, technically defined, does not refer or correspond to an external reality. (p. 199; emphasis added)

Barnes-Holmes’s assertion that the position he took (and that closely resembles Skinner’s) is “a-ontological” is critical, because that implies that behavior analysts can adopt Skinner’s radical behaviorism to underpin their scientific practice without adopting a particular position on the nature of reality.

My overall aim is to articulate metaphysical assumptions likely to be held by behavior analysts, rather than change them, but in so doing to make clear some differences between those assumptions and those implicit in the approach of others in the Western tradition, whether philosophers, psychologists, or neither. As with other sciences, behavior analysis has discipline-specific assumptions (see Leslie, 2019) but I will not review those here. Instead, I consider whether there are fundamental assumptions, based on what we seem to know a priori, that we bring to behavior analysis but do not usually consider within the discipline.

## Strawson’s Naturalism and Behavior Analysis

I am going make an excursion into analytic philosophy because this branch of philosophy seems the closest to behavior analysis. In a broad sense, analytic philosophy is characterized by an emphasis on argumentative clarity and precision, often making use of formal logic and conceptual analysis, and in its heyday was much concerned with how an analysis of language can explain how we think (see Føllesdal, 1996, for an extended discussion of its definition). Gilbert Ryle’s philosophy of this type (e.g., Ryle, 1949) is familiar to many behavior analysts, and he was succeeded in his chair at Oxford University by P. F. Strawson (1959, 1966), and over a long career Strawson discussed many topics of relevance to behavior analysis. Strawson (1985) noted that there is an important tradition of skepticism in philosophy, and that skepticism is not the denial of any statements or beliefs that others take to be self-evident, but rather the assertion that these statements or beliefs cannot be proved to be true. There are four traditional targets of philosophical doubt:

1. The existence of the physical world.
2. Our knowledge of other minds.
3. The justification of induction.
4. The reality of the past.

Of these (1) is perhaps the most important, because the others lose much significance if the physical or external world does not exist. Before considering some philosophical arguments, I will comment on these statements from the perspective of behavior analysis. I have suggested elsewhere (Leslie, 2015) that in general behavior analysts are naïve realists (and I noted earlier that this is not inconsistent with the pragmatic stance typical of behavior analysis), in that we take for granted that the external world exists, and this is a belief we share with virtually everybody else (but see Baum [2017] for an alternative view). What about the other three targets? Well, I think that we are firmly committed to our own versions of (3) and (4). In a classic discussion of induction, Russell (1912) gives the example of the chicken that ran across to the yard to be fed by the farmer every morning, until the last day of its life when the farmer wrung its neck rather than feeding it. It turned out that the appearance of the farmer did not infallibly signal feeding time for the chicken, and we have had a lot to say about how this type of induction about regularities in the world influences behavior. In general, a history of reinforcement strongly influences current behavior, but does not guarantee that previously reinforced behavior will continue to be adaptive. Likewise, we not only believe in the reality of the past but have assembled many data about the relationships between events in the past and what will happen now. Recent behavior-analytic research and theory have shown that the experienced structure of past environments determines the choices made of future behavior (Cowie & Davison, 2020). That leaves (2): where do we stand on the problem of other minds?

For us, this gets confounded with the problem of minds in general, and consideration of both is helped by looking at some philosophical issues. Strawson (1985) takes us back to the strand of “naturalism” in David Hume’s philosophy. In Strawson’s words, Hume claimed that, “We simply *cannot help* believing in the existence of body, and *cannot help* forming beliefs and expectations in general accord with the basic canons of induction. He might have added that the belief in the existence of other people (hence other minds) is equally inescapable” (Strawson, 1985, pp. 8–9). According to Hume, these aspects of our thoughts come from “nature” not “reason,” and reason thus operates within bounds that are already set. Strawson recognized that this is dangerous territory for analytic philosophy because it involves accepting the limits of analysis and opens the possibility of many and various principles being asserted as fundamental and beyond challenge, but he was inclined to accept that we cannot help believing in certain things, and these include the four traditional targets of philosophical doubt. In looking for support for this position he was swayed by the enormous volume and complexity of scientific data that implies, without finally proving, the reality of the external world—but he also looked for philosophical support, and commended the work of Canadian philosopher Barry Stroud. Stroud (1979) reminded us that the skeptical argument is that subjective experience could be the way it is, without it being the case that physical or material things actually exist, and he then looked to see whether we can “defuse” or make “impotent” the skeptical argument, because he does not think we can disprove it in the conventional sense.

In a later article, Stroud (2000) summarized Immanuel Kant’s approach, which sought to prove what is now called a transcendental argument for the “synthetic a priori,” or factual statements that must be true if we are able to think at all about experience. If Kant’s approach (or a weaker version of it) succeeds, then, “the propositions that scepticism claims we can never know [will be] shown to be necessary conditions of our having the very thoughts and beliefs we need in asking the epistemological question of whether we can know such things” (p. 211). Stroud deduced various premises, or built-in assumptions, we must make if such thinking is in general to be possible. These include that there are people other than ourselves that have thoughts and beliefs, and that people in general believe that there are “enduring particulars,” that is, objects outside of ourselves that persist in space and time. Stroud argued that we may be able to show a type of invulnerability of these premises, because “we could never see ourselves as holding the beliefs in question and being mistaken. We could not consistently find that human beings are simply under the misapprehension or the illusion that those things are true” (p. 218).

So, it seems there are philosophical arguments to support the notion that we bring some unshakeable presuppositions about reality to our consideration of any science, including behavior analysis. Is there evidence that behavior analysts, through their writings explicitly or implicitly accept the existence of the physical world and knowledge of other minds (as well as induction and the reality of the past)? Both Hocutt (1977), approvingly, and Zuriff (1980), less so, discussed Skinner’s naturalistic ethics, mainly as explicated in *Beyond Freedom and Dignity* (Skinner, 1971). Although “naturalism” comes in various forms, not necessarily identical with Strawson’s version, Zuriff (p. 347) identified a quotation from an earlier work of Skinner’s that does seem germane here: “Do not ask me why I want mankind to survive. I can tell you why only in the sense in which the physiologist can tell you why I want to breathe” (Rogers & Skinner, 1956, p. 106S). Discussing this, and another longer quotation on the same theme, Zuriff concluded that Skinner’s view was that

because of a specific phylogenetic and cultural history, individuals evolve for whom group survival will eventually emerge as an explicit prior condition for cultural design. This will happen not because a value is explicitly chosen but because, *given human nature*, people will simply behave this way. (p. 348; emphasis added)

Although in the words of a sympathetic commentator rather than Skinner, the reference to human nature echoes Strawson’s gloss on Hume cited earlier.

## Rorty and Wittgenstein’s Alternatives to Dualism

I now consider some ideas of the American philosopher, Richard Rorty. Although a version of naturalism is one strand of his overall approach (Rorty, 1979, p. 373), Rorty’s view was that accepting a Strawsonian naturalism inevitably also leads to what we might call mentalism or dualism, primarily because Strawson retains the Kantian distinction between “intuitions,” items of which we are aware without giving them any description often described as sense data by philosophers, and concepts that describe or classify those items (see Rorty, 1970). However, contra Strawson, he did not think that we should accept this type of naturalism as inescapable. In his important work, *Philosophy and the Mirror of Nature*, Rorty (1979) maintained that all apparently indisputable philosophical assumptions should be seen in their historical context. He espoused a type of pragmatic relativism whereby we must find arguments that work for us in our own historical context, but not expect our analysis to reveal immutable truths about reality or ourselves.

He identified several ideas that have emerged in different eras but have remained with us, usually unconsidered. One of these arose with the ancient Greeks, who for example dealt with the difference between knowing that there were parallel mountains to the West and knowing that infinitely extended lines never meet, by saying that the body sees mountains, but the so-called eye of the mind sees such universals, and this capacity distinguishes humans from other animals. Rorty notes that,

There was, we moderns might say with the ingratitude of hindsight, no particular reason why this ocular metaphor seized the imagination of the founders of Western thought. But it did, and contemporary philosophers are still working out its consequences, analyzing the problems it created, and asking whether there might not be something to it after all. (p. 38)

As well as pointing out problems arising from accepting the notion of an eye of the mind that somehow inspects immaterial things, Rorty noted that over time different types of entities have been said to be “mental.” He classified these, with some being phenomenal and representational, such as thoughts and mental images, whereas some are not phenomenal but are representational, such as beliefs and desires, and some are phenomenal but not representational, such as pains or other “raw feels.” Furthermore, Rorty provided a list of nine ways in which beings with minds are said to be different from beings without minds. He called these the possible “marks of the mental”:

1. The ability to know itself incorrigibly (or have “privileged access”).
2. The ability to exist separately from the body.
3. Having a nonspatial part or element.
4. The ability to grasp universals.
5. The ability to sustain relations to the inexistent (“intentionality”).
6. The ability to use language.
7. The ability to act freely.
8. The ability to form part of our social group (be “one of us”).
9. The inability to be identified with any worldly object.

This is a long and diverse list, and perhaps there is no true account of reality and our relation to it lurking here. Rather, it may just be that different cultures have thrown up different accounts of the key differences between “man” and “brutes” (the issue that Watson [e.g., Watson, 1930] realized preoccupied 19^th^-century psychology, and that he was so keen to set aside), or, in modern terms, the search for human uniqueness. Rather, perhaps as Rorty maintained, we should find arguments that work for us in our own historical context.

As an example, consider one long-standing debate between behaviorists and “dualists.” A familiar idea is that the mind “mirrors” and “represents” nature or reality. This notion developed in the 17^th^ century but is culturally still with us, and generates many problems that, according to Rorty, we can escape if we drop the presuppositions of naturalism. The debate was summarized by Donagan (1966) as follows. The dualist or Cartesian asserts: “My sensations are private, so no-one else can know whether I have a specific pain.” But, Donagan said, it follows from that that when I see a child pour boiling water on herself and then scream and writhe in pain, I am making a dubious inference in saying that she is in pain, which is absurd. On the other hand, a behaviorist might claim, “To say ‘She is in pain,’ asserts nothing more than what can be observed from her behavior and environmental circumstances.” But, Donagan maintained, it follows from that that if I say, “I am in pain,” I say nothing more than you can observe, perhaps better than me, which is also absurd. Donagan and Rorty preferred Wittgenstein’s (1953) alternative: “*I* cannot be said to learn of [my sensations]. I *have* them” (p. 89). Thus, pains can be sensations, without being overt behavior or immaterial things observed by the mind’s eye.

Wittgenstein’s alternative to dualism is spelt out in much more detail in his posthumous work, *On Certainty* (Wittgenstein, 1972). Like much of his later work, this consists of many statements, not all apparently consistent with each other, rather than a linear narrative, but Moyal-Sharrock (2004) provided a lucid exposition that addresses many of the questions raised here:

According to [such] Ghost-in-the-Machine philosophers, we think the thoughts we do and perform the acts we do *because* of some prior internal cognitive processing. One of Wittgenstein’s greatest contributions to philosophy is to have shown that, in some cases, positing a cognitive process antecedent to our thoughts and actions is misguided, redundant (idle) and misleading. (p. 203)

I began by citing the key statements or beliefs that are subjected to analysis by skeptics, but a key idea of Wittgenstein’s was that of the “hinge belief” or “hinge certainty”:

Hinge beliefs are certainties whose verbal articulation for heuristic purposes deceives us into thinking that we have here to do with propositional beliefs. In fact, their nonreflective or animal nature make them an imperceptible bridge where there once seemed an incomprehensible gap between our thinking and our acting. (Moyal-Sharrock, 2004, pp. 10–11)

Much of *On Certainty* is spent developing and defending the notion that such hinges are indeed not propositions that can be expressed in language and accepted or rejected on the basis of rational argument. According to Moyal-Sharrock (2004, p. 72), Wittgenstein’s hinges all have these properties. They are: (1) indubitable: doubt and mistake are logically meaningless; (2) foundational: they do not result from justification; (3) nonempirical: they are not derived from the senses; (4) grammatical: they are rules of grammar; (5) ineffable: they cannot be said; (6) enacted: they can only show themselves in what we say and do. On Wittgenstein’s analysis, to believe something may on occasion be just be acting in a certain way; beliefs can be said or shown. Moyal-Sharrock concluded that,

*On Certainty* allows us to extend our conception of belief to include a nonpropositional, nonmental, enacted occurrence of belief, thereby avoiding the pseudoproblem of connecting propositional belief to action. It shows us that the occurrence of a belief is not necessarily a mental manifestation, but can also be a physical manifestation, and that it is therefore not *necessary* that a belief be “realised in” a physical state in order to move us to action. Hinges are not ghosts in the machine, but part of the machinery—perhaps the oil without which the machine cannot work smoothly and automatically. (pp. 203–204)

## Neutral Monism as a Framework for Behavior Analysis

I have suggested that we, as Western scientists, are typically naïve realists and implicitly accept some form of naturalism. I have then outlined several strands of philosophical analysis that indicate that the dualism inherent in many forms of naturalism can be avoided without changing that general philosophical stance. If, as behavior analysts, we are not dualists, then, in terms of philosophical classification, we are monists, monism being the view that denies the existence of a distinction or duality in a particular sphere, such as that between matter and mind. Monism is in turn a philosophical position that has to be developed beyond the simple rejection of dualism. Clavijo-Alvarez (2019) suggested that we should be neutral monists, neither treating everything as material nor everything as mental, and could usefully adopt the framework developed by William James. James’s approach was close to that of contemporary ecological psychology as well as behavior analysis in that it was explicitly antirepresentational. James (1912) stated his fundamental ontological assumption (to use Clavijo-Alvarez’s [2019] term) in the following way:

My thesis is that if we start with the supposition that there is only one primal stuff or material in the world, a stuff of which everything is composed, and if we call that stuff pure experience, then knowing can easily be explained as a particular sort of relation towards one another into which portions of pure experience may enter. (p. 4)

James considered that other philosophers had generated representational theories because they assumed that only individual elements of the world could be directly perceived, but he denies that this is the case: “The statement of fact is that the relations between things, conjunctive as well as disjunctive, are just as much matters of direct particular experience, neither more so nor less so, than the things themselves” (James, 1909, p. 280).

Banks (2014) noted that what James called “pure experiences” were called “elements” by Mach (1886), and “event particulars” by Russell (1921) in similar accounts of neutral monism, and these accounts can be summarized in four principles:

1. Monism: the mental and physical domains are part of a greater natural domain of elements and their functional variations.
2. Neutralism: elements are neither mental nor physical; rather minds and physical bodies are functionally related complexes of elements with no underlying duality.
3. Psychophysical Identity: every sensation, such as green, is also a physical element, a neural energy in the brain. Not every element is a sensation, or even a possible sensation.
4. Powers: elements are powers with causal force. Every element is embedded in its functional role (Banks, 2010, p. 175).

Adopting such a framework does have implications beyond setting aside discussions of dualism. One is that it suggests a framework within which to approach consciousness by implying some form of panpsychism. There are many forms of this notion that any form of organic complexity gives to some degree of that which is called consciousness in humans (see Goff, Seager, & Allen-Hermanson, 2017), but most forms strengthen the case for phylogenetic continuity. Behavior analysts typically strongly endorse the importance of phylogenetic continuity in learning processes, a position that is finding growing support in evolutionary biology (Van Duijn, 2017).

## Wider Implications for Behavior Analysis

To summarize the argument so far: it may be necessary to review metaphysical assumptions rather than launching into scientific work without so doing; the naturalistic Western tradition seems to imply mentalism or dualism with all the attendant paradoxes; if, however, we take Rorty’s historicist view or accept Wittgenstein’s analysis, we can as behavior analysts adopt the neutral monist alternative that developed within 20^th^ century. Articulating this nondualist framework will encourage links with ecological psychology (Reed, 1996), indeed the quotation from James (1909) resembles later statements by J. J. Gibson (1979) and may persuade behavior analysts to provide a more coherent account of perception and memory (Tonneau, 2011). It may also provide us with a narrative with which to engage the vast regiment of cognitive psychologists (or cognitivists, more broadly) who tend to reject our approach out of hand on conceptual grounds without reflecting on the flaws in their own approach. Schlinger (2004, 2018) was able identify a number of statements by contemporary cognitive psychology that clearly imply a type of dualism. He concluded,

These statements seem to reflect what is called property (vs. substance) dualism—the view that while the world consists of only one substance—physical events—there can be different properties of that substance. According to property dualists, mind and behavior represent two different properties of one substance: the brain. (Schlinger, 2018, p. 164)

Does this sketch of some issues, and a cautious recommendation of neutral monism, help us resolve any debates within behavior analysis? One possibility concerns the status of private events. Here are two authoritative position statements. Moore (2011) wrote,

Although many behavioral events are publicly observable, not all are. Some behavioral events are "private," in the sense that they are accessible to only the person who is behaving. Private behavioral events are determined by the tools or characteristics of an observer, rather than by anything pertaining to the nature of the event itself. . . . [T]hese private behavioral events gain their functional significance through public relations. Consequently, there is no appeal to autonomous private entities from another dimensional system as causes of behavior. Behavioral principles are developed through the analysis of public behavior and then used in interpretations of private forms. (p. 457)

Although Baum (2011) wrote,

Many different types of private events occur within the skin: neural events, events in the retina, events in the inner ear, subvocal speech (i.e., thinking), and so on. All of these are possibly measurable and, therefore, possibly public. I will argue that private events are not useful in a science of behavior, and, far from being a key defining aspect of radical behaviorism, private events constitute an unnecessary distraction. Private events are irrelevant to understanding the function of behavior, that is, activities in relation to environmental events. Because the origins of behavior always lie in the environment, the origins of behavior are public. (p. 186)

The second of these quotations is part of an acrimonious debate between the two which covered disagreements on other topics in behavior analysis but primarily focused on the status of private events. A common theme of both accounts is observability and its limits. The term “observation” typically occurs in definitions of science before a rapid shift from that term to practical matters of measurement. We are aware that our capacity to observe and measure is changing dramatically, in particular where the subject matter is human behavior, and this has scientific, clinical, social, and political implications (Zuboff, 2019). In the behavior analysis debate about private events, we are mostly concerned with the limits of observation of an individual’s behavior. Both authors stated that some activities are public whereas others are not. The difference is that Moore said that it is useful to extrapolate our framework based on public behavior and environmental events to private behavior, whereas Baum said that this is counterproductive and runs the risk of undermining the antidualism position, which is fundamental to radical behaviorism and behavior analysis. A partial solution may be to abandon the public/private distinction, which we know is shifting for purely technological reasons. From the point of view of the neutral monist-realist framework outlined earlier, we would say that many behaviors occur in the world, but from the perspective of behavior analysis those that are shown to be functional (for the individual) are the ones of interest.

The argument perhaps then boils down to whether it is useful to speculate about aspects of behavior and environment–behavior relations that we cannot currently observe. Baum stated that it is unnecessary and dangerous, Moore claimed it to be useful and illuminating (and of course there are other scientists, not cited here, on each side of the argument). This is perhaps not a large gap and can be seen as a difference in strategic standpoint. It is regrettable that the debate has generated a lot of disagreement within a field where we are all pragmatists, concerned with finding out what works. In a pragmatic sense, it would be more helpful if we identified ways of sharing more explicitly our conceptual framework within the field while concurrently engaging in a more productive discussion with nonbehaviorists. In looking outwards and talking to those who fundamentally misunderstand our approach, there will be occasions when it is effective to explain how our approach can be usefully extended to analyzing behavior not currently observable, and there will be occasions when it is effective to provide explanations of behavioral phenomena by looking at behavior–environment relations over longer time frames.

## Data Availability

N/A
